# A tool to make reporting checklists work

**DOI:** 10.1186/s12916-015-0476-3

**Published:** 2015-09-28

**Authors:** Ana Marušić

**Affiliations:** Journal of Global Health, Split, Croatia; Department of Research in Biomedicine and Health, University of Split School of Medicine, Split, Croatia

**Keywords:** CONSORT, Online tools, Reporting guidelines, Writing

## Abstract

Although the use of reporting guidelines has been demonstrated to increase the completeness and transparency of health research published in journals, there is still a long way to translate their use to the authors at the time where they are needed – during the actual research process and manuscript writing. An online tool for writing methodology section of a randomized controlled trial has been successfully tested in an experimental setting and provides a direction for the development of writing tools for health research. Writing tools should not replace original thinking and the excitement of communicating original discoveries, but make sure that all relevant data are in the manuscript so that research results can be understood, critically evaluated and used in practice.

Please see related article: http://www.biomedcentral.com/1741-7015/13/221

## Background

I am a physician by training, and a journal editor by chance. Both of these professions require discipline and use of aids, such as checklists, to compensate for the limits of human memory and attention and to prevent errors (in patient treatment or manuscript management). This is the reason why I read with enthusiasm the recent article by Barnes et al. in *BMC Medicine* [1], who performed a randomized controlled trial (RCT) to test an online tool for writing methods section of RCT articles. Primarily based on the 2010 CONSORT reporting guideline [2] and its explanatory document [3], the tool was tested in a sample of masters and doctoral students in public health and significantly increased the completeness of reporting for most of the methodological domains in an RCT report compared to a classical writing exercise.

While the previous studies demonstrated that CONSORT endorsement by journals was successful in increasing the completeness of trial reporting in medical journals [4], the study of Barnes et al. [1] is the first study to test the tool where and when it should be used – by researchers at the time of manuscript writing.

Considering that the first version of the CONSORT guideline was published almost 20 years ago [5] and is currently endorsed by more than 600 journals and most influential editorial organizations [6], why did it take so long to translate CONSORT into the actual practice of writing health research?

## The long road to checklist implementation in healthcare

Checklists made their way into medicine from industry, where they have been used as quality and safety assurance of processes and products, especially those carrying high risk [7]. The most popular and globally relevant example of a medical checklist is the WHO Surgical Safety Checklist, which was created in 2008 to reduce the rate of major surgical complications. It was tested simultaneously in eight hospitals around the world to demonstrate a highly significant reduction in complication or death rates after surgery [8]. In 2014, a systematic review of seven studies testing the WHO Surgical Safety Checklist demonstrated its consistent effect on the reduction of postoperative complications and mortality [9]. In experimental settings, checklists have also been proven as an effective tool to improve adherence to best practices during operating-room crises [10].

In the case of the WHO Surgical Safety Checklist, the evidence base for the checklist implementation built up quickly, but the implementation is still burdened by a number of barriers at the organizational, systems, team, or checklist-specific levels, as demonstrated by a qualitative evaluation of its nation-wide implementation in UK hospitals [11]. A recently published systematic review of qualitative evidence for barriers and facilitators of surgical safety checklists showed that the complex reality of healthcare practice requires approaches that go beyond barriers and facilitators to fostering teamwork, mutual understanding, and communication [12].

## The complex world of reporting guidelines

Currently, the most comprehensive source of information about reporting guidelines – the Enhancing the QUAlity and Transparency Of health Research (EQUATOR) Network, lists 281 different reporting guidelines [13]. While only a few cover most common study designs in health research, such as CONSORT for RCTs, STROBE for observational studies, and PRISMA for systematic reviews, most are guidelines for specific study types or variations of standard methodology. Nevertheless, they all aim to improve the completeness and clarity of published research and thus reduce waste in research informing health practice [14]. Guidance is also available for developers of reporting guidelines [15].

However, the primary users of reporting guidelines should be the researchers and authors, who may find their use unavoidable and horrifying at the same time. On the one hand, they have to meet the expectations of journals about reporting guidelines. On the other, they may not be sure which reporting guideline to choose (for example, CONSORT has 10 current official extensions) and how to follow it: a checklist may have over 20 items [2], many of which are difficult to understand for an average clinical researcher without good knowledge of clinical epidemiology, while the “Explanation and Elaboration” documents sometimes have over 30 pages [3].

Nevertheless, the main problem is that the reporting guidelines are used too late in the research process, when the study has already been performed or sometimes even after the provisional acceptance of the manuscript. At that time, it may be too late to discover that the important things have been missed or could have been done better in order to increase the quality of the publication. My experience as a journal editor and teacher of research methodology to graduate and postgraduate medical students, residents, and physicians is that knowledge about reporting guidelines should be acquired at the graduate level, during the medical curriculum [16]. This is in line with observations from other seasoned clinical trialists, such as Dr. Thomas Chalmers, a physician with a pivotal role in the scientific development of the RCT and meta-analysis in the USA, who stated, “[i]*n medical school, I think we have to just hammer away at evidence and probability theory and general statistics*” [17], as well as recommendations from the International Society for Evidence-Based Health Care [18]. When medical or healthcare students learn critical appraisal and understanding of evidence early in the curriculum and as seriously as they do for any other medical course, they will be better practitioners, making better decisions with their patients as well as in performing and publishing research.

## Will writing tools for reporting checklists work?

My answer to the above question is – yes, writing tools will work. Examples of good practice are already there for healthcare researchers. Researchers working on Cochrane systematic reviews use Review Manager (RevMan) – the online tool that guides authors in preparing the text of the review, building tables, performing meta-analyses, and graphically presenting the results. The most recent development is RevMan HAL, a text-editor extension for RevMan developed by the Cochrane Schizophrenia Group, which helps authors to generate parts of the review automatically [19]. It has already been used to construct a first draft of review sections [20].

In clinical practice, the use of natural language generation system did not seem like a ready solution for automatic text generation of clinical reports in 2003 [21], but in 2013, computer-generated patient history summaries seem to be at least as accurate as records produced by clinicians while requiring less time to produce [22]. Of course, there is always a possibility of misuse of technology, as demonstrated by examples of computer-generated gibberish papers accepted at conferences [23], but this is a more complex problem of research and publication integrity [24].

## Conclusions

It is good to see that the efforts in providing assistance to increase the clarity and transparency of reporting health research have moved from journals to authors. The effectiveness of the writing tool needs to be tested in the real world – when and where research occurs. The tool should be further developed to be easy to use in all research settings, both in the developed and developing world. Finally, it should not replace original thinking and the excitement of communicating original discoveries, but make sure that all relevant data are in the manuscript so that research results could be understood, critically evaluated, and used in practice.

## References

[1] Barnes C, Butron I, Giraudeau B, Porcher R, Altman DG, Ravaud P. Impact of an online writing aid tool for writing a randomized trial report: the COBWEB (Consort Based WEB tool) randomized trial. BMC Med. 2015;13:221. 10.1186/s12916-015-0460-y PMC4570037 26370288

[2] Schulz KF, Altman DG, Moher D, for the CONSORT Group (2010). CONSORT 2010 Statement: updated guidelines for reporting parallel group randomised trials. BMC Med..

[3] Moher D, Hopewell S, Schulz KF, Montori V, Gøtzsche PC, Devereaux PJ (2010). CONSORT 2010 explanation and elaboration: updated guidelines for reporting parallel group randomised trials. J Clin Epi..

[4] Turner L, Shamseer L, Altman DG, Weeks L, Peters J, Kober T (2012). Consolidated standards of reporting trials (CONSORT) and the completeness of reporting of randomised controlled trials (RCTs) published in medical journals. Cochrane Database Syst Rev..

[5] Begg C, Cho M, Eastwood S, Horton R, Moher D, Olkin I (1996). Improving the quality of reporting of randomized controlled trials. The CONSORT statement. JAMA..

[6] Consolidated Standards of Reporting Trials. Impact of CONSORT. http://www.consort-statement.org/about-consort/impact-of-consort. Accessed 29 Aug 2015.

[7] Gawande A (2009). The checklist manifesto: how to get things right.

[8] Haynes AB, Wieser TG, Berry WR, Lipsitz SR, Breizat AH, Dellinger EP (2009). A surgical safety checklist to reduce morbidity and mortality in global population. New Engl J Med..

[9] Bergs J, Hellings J, Cleemput I, Zurel Ö, De Troyer V, Van Hiel M (2014). Systematic review and meta-analysis of the effect of the World Health Organization surgical safety checklist on postoperative complications. Br J Surg..

[10] Arriaga AF, Bader AM, Wong JM, Lipsitz SR, Berry WR, Ziewacz JE (2013). Simulation-based trial of surgical-crisis checklists. N Engl J Med..

[11] Russ SJ, Sevdalis N, Moorthy K, Mayer EK, Rout S, Caris J (2015). A qualitative evaluation of the barriers and facilitators toward implementation of the WHO surgical safety checklist across hospitals in England: lessons from the “Surgical Checklist Implementation Project”. Ann Surg..

[12] Bergs J, Lambrechts F, Simons P, Vlayen A, Marneffe W, Hellings J (2015). Barriers and facilitators related to the implementation of surgical safety checklists: a systematic review of the qualitative evidence. BMJ Qual Saf.

[13] EQUATOR Network Search for reporting guidelines. http://www.equator-network.org/reporting-guidelines/. Accessed 29 Aug 2015.

[14] Glasziou P, Altman DG, Bossuyt P, Boutron I, Clarke M, Julious S (2014). Reducing waste from incomplete or unusable reports of biomedical research. Lancet..

[15] Moher D, Schulz KF, Simera I, Altman DG (2010). Guidance for developers of health research reporting guidelines. PLoS Med..

[16] Marušić A, Sambunjak D, Jerončić A, Malički M, Marušić M (2013). No health research without education for research–experience from an integrated course in undergraduate medical curriculum. Med Teach..

[17] Maclure M (1996). Dr. Tom Chalmers, 1917–1995: Trials of a randomizer. CMAJ..

[18] Glasziou PP, Sawicki PT, Prasad K, Montori VM, International Society for Evidence-Based Health Care (2011). . Not a medical course, but a life course. Acad Med..

[19] Cochrane Schizophrenia Group. RevMan HAL v 4.0 Frequently asked questions. http://szg.cochrane.org/revman-hal-v-40-frequently-asked-questions. Accessed 29 Aug 2015.

[20] Morris NR, Kermeen FD, Holland AE (2014). Exercise-based rehabilitation programmes for pulmonary hypertension (Protocol). Cochrane Database Syst Revs..

[21] Hüske-Kraus D (2003). Text generation in clinical medicine – a review. Methods Inf Med..

[22] Scott D, Hallett C, Fettiplace R (2013). Data-to-text summarisation of patient records: using computer-generated summaries to access patient histories. Patient Educ Couns..

[23] Van Noorden R. Publishers withdraw more than 120 gibberish papers. Nature News, 24 Feb 2014. http://www.nature.com/news/publishers-withdraw-more-than-120-gibberish-papers-1.14763. Accessed 31 Aug 2015.

[24] Stahel PF, Moore EE (2014). Peer review for biomedical publications: we can improve the system. BMC Med..

